# Large-scale analysis of interobserver agreement and reliability in cardiotocography interpretation during labor using an online tool

**DOI:** 10.1186/s12884-024-06322-4

**Published:** 2024-02-14

**Authors:** Imane Ben M’Barek, Badr Ben M’Barek, Grégoire Jauvion, Emilia Holmström, Antoine Agman, Jade Merrer, Pierre-François Ceccaldi

**Affiliations:** 1 Service de Gynécologie Obstétrique, Assistance Publique Hôpitaux de Paris – Hôpital Beaujon, 100 boulevard du Général Leclerc, Clichy La Garenne, France; 2 Present Address: Université Paris Cité, 75006 Paris, France; 3 Health Simulation Department, iLumens, Université Paris Cité, Paris, France; 4 Genos Care, Paris, France; 5 AP-HP.Nord-Université Paris Cité, Hôpital Universitaire Robert Debré, Unité d’épidémiologie clinique, 1426 InsermParis, CIC France; 6 Service de Gynécologie-Obstétrique et Médecine de la reproduction, Hôpital Foch, 40 Rue Worth, 92150 Suresnes, France

**Keywords:** Cardiotocography, Fetal heart rate, Interobserver agreement, Fetal hypoxia, Intrapartum, Labor

## Abstract

**Background:**

While the effectiveness of cardiotocography in reducing neonatal morbidity is still debated, it remains the primary method for assessing fetal well-being during labor. Evaluating how accurately professionals interpret cardiotocography signals is essential for its effective use. The objective was to evaluate the accuracy of fetal hypoxia prediction by practitioners through the interpretation of cardiotocography signals and clinical variables during labor.

**Material and methods:**

We conducted a cross-sectional online survey, involving 120 obstetric healthcare providers from several countries. One hundred cases, including fifty cases of fetal hypoxia, were randomly assigned to participants who were invited to predict the fetal outcome (binary criterion of pH with a threshold of 7.15) based on the cardiotocography signals and clinical variables. After describing the participants, we calculated (with a 95% confidence interval) the success rate, sensitivity and specificity to predict the fetal outcome for the whole population and according to pH ranges, professional groups and number of years of experience. Interobserver agreement and reliability were evaluated using the proportion of agreement and Cohen’s kappa respectively.

**Results:**

The overall ability to predict a pH level below 7.15 yielded a success rate of 0.58 (95% CI 0.56-0.60), a sensitivity of 0.58 (95% CI 0.56-0.60) and a specificity of 0.63 (95% CI 0.61-0.65). No significant difference in the success rates was observed with respect to profession and number of years of experience. The success rate was higher for the cases with a pH level below 7.05 (0.69) and above 7.20 (0.66) compared to those falling between 7.05 and 7.20 (0.48). The proportion of agreement between participants was good (0.82), with an overall kappa coefficient indicating substantial reliability (0.63).

**Conclusions:**

The use of an online tool enabled us to collect a large amount of data to analyze how practitioners interpret cardiotocography data during labor. Despite a good level of agreement and reliability among practitioners, the overall accuracy is poor, particularly for cases with a neonatal pH between 7.05 and 7.20. Factors such as profession and experience level do not present notable impact on the accuracy of the annotations. The implementation and use of a computerized cardiotocography analysis software has the potential to enhance the accuracy to detect fetal hypoxia, especially for ambiguous cardiotocography tracings.

**Supplementary Information:**

The online version contains supplementary material available at 10.1186/s12884-024-06322-4.

## Introduction

Hypoxia is a leading cause of neonatal morbidity and mortality. It can have consequences such as hypoxic-ischemic encephalopathy (HIE), organ dysfunction, developmental delays or cognitive impairments impacting the overall development of the baby [1]. Prompt medical intervention is crucial to minimize the potential long-term effects and improve outcomes for affected infants. Cardiotocography (CTG) is a non-invasive device that records fetal heart rate (FHR) and uterine contractions (UC). It is widely used as a screening tool in obstetric practice to determine fetal wellbeing. Specifically, obstetricians and midwives employ it during labor to identify fetal hypoxia, enabling them to intervene promptly in case of a pathological signal.

CTG analysis and interpretation is performed visually by obstetricians and midwives following guidelines [2]. There are different classifications with varying characteristics [2–6], without clear international consensus among them [7–10]. Although the guidelines are constantly being challenged and reviewed [11, 12], the overall process of interpreting CTG during delivery is known to be subjective and to induce a significant interobserver and intraobserver variability [13, 14].

The primary constraints of studies examining the interobserver and intra-observer variations are the limited number of both the assessors and the annotated cases [15]. Therefore, to answer these limitations, we have developed a tool available at www.fhr-annotator.com that facilitates practitioners in annotating 100 cases sourced from the CTU-UHB open database [16]. The objectives were to evaluate the accuracy of fetal hypoxia prediction and interobserver agreement and reliability among a wide range of practitioners using an open-source database with CTG signals, clinical data and fetal outcomes.

## Methods

We have built our methodology based on the Guidelines for Reporting Reliability and Agreement Studies (GRRAS) [17].

### Description of the survey

We performed a cross-sectional online survey inviting clinicians to predict the fetal outcome based on CTG signals and clinical variables. We have built a publicly available web tool written in Python and compatible with most modern web browsers [18]. At the start of the study period, we shared the website widely via email through medical associations, and directly to heads of obstetrics and gynecology departments. The survey took place from November 2022 to January 2023. When browsing the website for the first time, the participant was asked to create an account by providing the following information: an anonymous and unique identifier, age, profession (obstetrician, resident, midwife or student midwife), number of years of experience (since diploma for non-students), place of practice (university hospital or general hospital) and country. We have considered asking additional information to the participants (for example whether they did specific trainings on CTG interpretation, they use scalp samples in clinical practice, and which CTG interpretation guidelines they use), but we decided to limit the number of information requested to simplify the use of the tool.

One hundred cases were displayed successively to the participant for annotation (Figure S1). The available information was the FHR and UC signals in the last 45 minutes before delivery and some relevant clinical information (sex, term of gestation and neonatal weight). We choose to use minimal clinical information to simplify the annotation process and to have as much labelled cases as possible. The layout of the tool mimics a standard paper graph (standard scale of 1cm per minute). For every case, the user was asked to predict the fetal outcome (normal outcome or fetal hypoxia) and to draw the FHR baseline. Only the results concerning the fetal outcome prediction have been presented in the current paper. The cases were assigned to each participant in a pseudorandom order: the same batches of 10 cases (5 with a normal outcome and 5 with fetal hypoxia) were presented to all participants but with a random order inside each batch. This method ensured a higher proximity in the cases annotated by the different participants than drawing them randomly among the 100 available cases. The participants were free to label as many cases as they wanted until 100. Upon each validation, users were informed of the correctness of their fetal outcome prediction. They were able to log out of their account and to sign in again later using their identifier to continue annotating.

The 100 cases were selected from the CTU-UHB dataset [16], which contains CTG signals (FHR and UC) and clinical information of deliveries occurring between 2010 and 2012 at the University hospital in Brno, Czech Republic. FHR signals were obtained by external ultrasound transducer or by direct scalp electrode, depending on the cases. The database represented 506 vaginal deliveries (with 44 operative deliveries), including 89 cases with fetal cord sample pH lower than 7.15. The full description of the selection process and the maternal and neonatal characteristics have been previously published [16]. Briefly, it includes patients over 18 who delivered singleton term fetuses with a second stage of labor lasting less than 30 minutes. Fetuses with known intrauterine growth restriction, malformation or infection were excluded. To complete the database, all the cases were annotated by nine Czech obstetric experts (named “the CTU-UHB experts”, hereafter) who predicted the labor outcome (e.g. estimated pH result for neonatal hypoxia) based on CTG signals and some clinical characteristics [14]. We randomly chose 50 cases exhibiting a pH level below 7.15 (corresponding to a moderate fetal hypoxia) and 50 other cases with a pH level higher than 7.15 (corresponding to a normal outcome). The pH threshold of 7.15 was the one used to discriminate normal from abnormal neonatal outcome in the CTU-UHB database [14]. We have kept the same threshold to facilitate the comparisons with the CTU-UHB experts annotations and also because it has been previously shown that even in case of moderate hypoxia there is an increased risk of fetal complication [19].

Based on the sensitivity and specificity of the prediction of the CTU-UHB experts, we performed a sample size calculation to determine the number of FHR needed in our study. With a sensitivity of 0.45, a specificity of 0.67, a precision in 95% confidence interval of 0.14 and a pre-specified prevalence of mild hypoxia of 50%, we determined that 97 cases are needed to conclude [20]. We have rounded the number to 100 cases including 50 cases of mild fetal hypoxia.

### Outcomes

The primary outcome was the accuracy of the participants prediction. The secondary outcome was the interobserver agreement and reliability.

### Statistical analysis

First, we performed a descriptive analysis of the participants and their annotations. Continuous variables were described using median and quartiles [Q1-Q3] and qualitative variables using numbers and percentages.

We analyzed the accuracy of the predicted outcomes for each participant and case using success rate (defined as the ratio of correctly predicted cases to the total number of cases), sensitivity and specificity. All metrics were accompanied by their respective 95% confidence intervals, computed using the Wilson method, which is more reliable when dealing with extreme proportion or small sample sizes [21]. The evaluation was carried out comprehensively for all annotations and according to the participants’ profession (residents, obstetrician-gynecologists, midwives) and number of years of experience (0 - 2 years, 2 - 4 years, 4 - 8 years and >8 years). We considered that there was a significant difference in accuracy when the 95% confidence intervals of success rate, sensitivity or specificity overlapped. Additionally, to illustrate the relationship between the success rate and the pH value at birth, the average success rate was evaluated across distinct ranges of pH levels defined by the following thresholds: 6.90, 6.98, 7.05, 7.13, 7.20, 7.28, 7.35 and 7.43. The thresholds were selected to ensure a balanced distribution of cases across each range.

We have also made a graphical analysis of the participants’ performance and interobserver variability by plotting the true positive rate (TPR, or sensitivity) against the false positive rate (FPR, or 1-specificity) for each participant. The size of the point corresponding to a participant is proportional to the number of annotations for this participant. We only show the participants with more than 10 annotations on this plot. We have extracted the existing annotations of the 100 cases included in our study by the nine CTU-UHB experts [14], which enabled to position them on the plot.

We evaluated the agreement and reliability between the professions using the proportion of agreement (PA) [22, 23] and Cohen’s Kappa coefficient (κ) respectively [24], as recommended in the literature [17, 25]. Agreement measures whether users’ annotations are similar. Reliability, on the other hand, corresponds to the ratio of the variability between the annotations of the same cases to the total variability of all annotations. 95% confidence intervals (CI) on those metrics were calculated. PA is defined as the proportion of cases for which the participants agreed on, and the Altman classification system [26] was employed to categorize the findings as follows: 0.81 to 1.00 indicates very good, 0.61 to 0.80 indicates good, 0.41 to 0.60 is moderate, 0.21 to 0.40 is fair, and a value below 0.2 is considered as a poor interobserver agreement. Moreover, if the lower boundary of the 95% CI for PA fell below 0.50, the agreement level was also regarded as non-significant [22]. Kappa quantifies the similarity between two sets of categorical ratings, adjusting for the degree of overlap that could happen randomly. It ranges from -1 to 1 with the following predefined interpretations: a value below 0.20 indicates slight, 0.21 to 0.40 fair, 0.41 to 0.60 moderate, 0.61 to 0.80 signifies substantial, and a value above 0.80 as almost perfect reliability [23, 24]. As kappa is sensitive to the prevalence, a low prevalence of pathological cases in the cohort could lead to a kappa close to zero, even if there is a high observed PA between practitioners [17, 27]. In our analysis, the prevalence is 50% as we have drawn the same number of cases in both groups, which makes the two metrics comparable. To calculate these metrics, the participants were categorized by their declared professions. Within each profession, we considered for each case annotated by at least one participant the most frequent annotation as the profession's consensus. We then calculated the pairwise agreement and reliability between these consensus annotations, considering only the cases annotated by at least one participant in the two considered professions (overlapping cases). Finally, we derived an overall agreement measure by taking a weighted average of these pairwise values, where the weights reflected the number of overlapping cases for each pair of professions. This approach accounts for the varying contributions of different profession pairs to the overall agreement metric.

Statistical analyses were performed using Python along with its associated libraries (`scikit-learn` and `statsmodels` for statical analysis, `pandas` for data manipulation and `plotly` for visualization).

## Results

### Descriptive analysis of the participation (Table 1)

During the inclusion period there were 120 participants. Most of them were based in France (94), and 84% were affiliated with a university hospital. The participants were grouped per profession into 3 groups: residents [28], obstetrician-gynecologists (58) and midwives [22]. Only 2 participants identified as student midwives and they were aggregated with non-student midwives. A total of 2950 annotations were collected during the study, with a median of 11 annotations per participant (q1=5, q3=36). 12 participants annotated the 100 cases and 62 annotated more than 10 cases (Figure S2). The midwives exhibited the highest median participation with 16 annotations, followed by the obstetrician-gynecologists and residents with 9 annotations in both groups (Table 1).

**Table 1:** General information of participants

**Profession**	**General Information**		
	**Number of assessors (%)**	**total number of annotations (%)**	**Number of annotations per assessor Median (q1, q3)(%)**
Residents	39 (33)	885 (30)	9 (3, 29)
Midwives	23 (19)	826 (28)	16 (5, 65)
Obstetrician-Gynecologists	58 (48)	1239 (42)	9 (4, 28)
**Total**	**120 (100)**	**2950**	**11 (5, 36)**

### Accuracy of hypoxia prediction

**Fig. 1:**
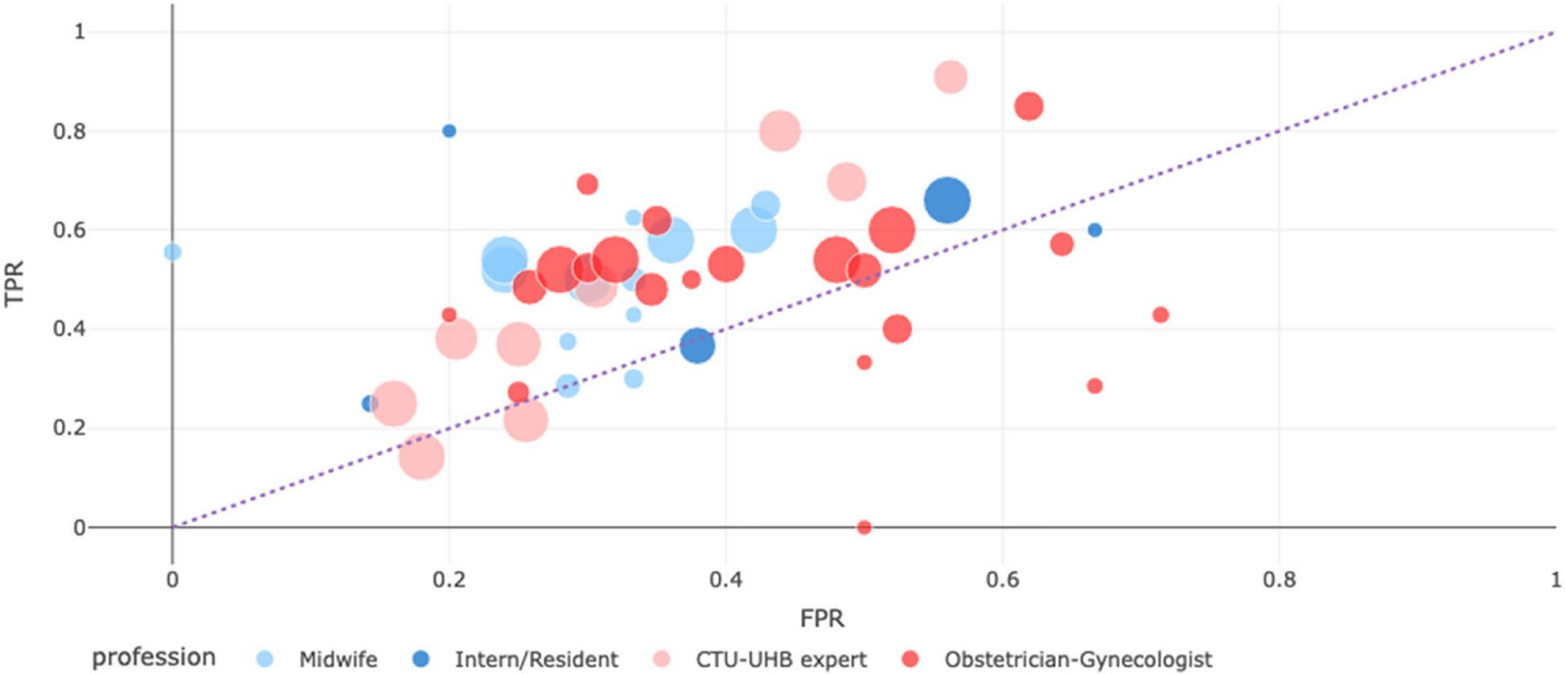
Graphical representation of the participants’ performance. TPR true positive rate, FPR false positive rate

The overall success rate, sensitivity and specificity were 0.58 (95% CI 0.56-0.60), 0.58 (95% CI 0.56-0.60) and 0.63 (95% CI 0.61-0.65) respectively (Table 2). The mean success rate in the different groups varied from 0.55 (obstetricians) to 0.61 (midwives), with no statistically significant difference observed between them. The success rate did not significantly depend on the number of years of experience (Figure S3): it was 0.59 (95% CI 0.55-0.64), 0.55 (95% CI 0.51-0.60), 0.58 (95% CI 0.54-0.62) and 0.55 (95% CI 0.51-0.60) in the 0 - 2 years, 2 – 4 years, 4 – 8 years and >8 years groups respectively. On the same 100 cases, the success rate of the nine CTU-UHB experts was 0.56 (95% CI 0.53-0.60), with no significant difference with our results. The sensitivity and specificity were 0.43 (95% CI 0.40-0.46) and 0.68 (95% CI 0.65-0.71) respectively, showing a lower specificity and higher specificity. Figure 1 illustrates those findings.

**Table 2:** Evaluation of fetal hypoxia prediction

**Profession**	**Evaluation of fetal hypoxia prediction**		
	**Success rate (95%CI)**	**Sensitivity (95%CI)**	**Specificity (95%CI)**
Residents	0.58 (0.54, 0.61)	0.56 (0.51, 0.60)	0.60 (0.55, 0.64)
Midwives	0.61 (0.58, 0.64)	0.54 (0.49, 0.59)	0.68 (0.63, 0.72)
Obstetrician-Gynecologists	0.55 (0.52, 0.58)	0.50 (0.46, 0.54)	0.61 (0.57, 0.64)
**Total**	**0.58 (0.56, 0.60)**	**0.58 (0.56, 0.60)**	**0.63 (0.61, 0.65)**

**Fig. 2:**
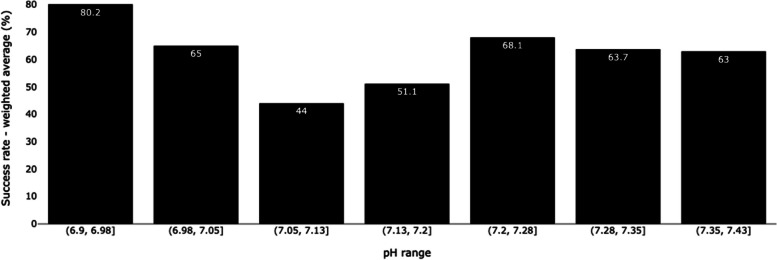
Mean success rate per pH range

Notably, the lowest success rate was obtained on signals around our pH threshold at 7.15: 0.44 for recordings within the 7.05-7.13 pH range, and 0.51 for recordings within the 7.13-7.20 range (Fig. 2). The highest success rate (0.80) was obtained on signals exhibiting pH values below 6.98. For signals with a pH value above 7.20, the success rate varied between 0.63 and 0.68.

### Interobserver agreement and reliability (Table 3)

We found a good agreement between participants (PA=0.82, 95% IC 0.68-0.96). The agreements between our different groups based on profession were similar (between 0.79 and 0.85). We found a substantial reliability among professionals (kappa=0.63, 95% IC 0.50-0.76), with similar values when evaluated per profession (between 0.58 and 0.68) Table 3.

**Table 3:** Interobserver proportion of agreement (PA) and reliability (Kappa coefficient)

**Profession**	**PA**				**Kappa’s coefficient**			
	**Residents (95%CI)**	**Midwives (95%CI)**	**Obstetrician-Gynecologists (95%CI)**	**Overall (95%CI)**	**Residents (95%CI)**	**Midwives (95%CI)**	**Obstetrician-Gynecologists (95%CI)**	**Overall (95%CI)**
Residents		0.79 (0.65, 0.93)	0.82 (0.68, 0.96)			0.58 (0.45, 0.70)	0.64 (0.51, 0.77)	
Midwives			0.85 (0.71, 0.99)				0.68 (0.55, 0.81)	
Obstetrician-Gynecologists								
**Total**				**0.82 (0.68, 0.96)**				**0.63 (0.50, 0.76)**

## Discussion

With 2950 annotated cases and 120 participants from different professions and experience levels, our study is the largest evaluating the accuracy and the interobserver variability of CTG interpretation during labor. Over the whole set of annotations, we found a moderate mean success rate (0.58) in predicting fetal hypoxia, and the sensitivity and specificity were 0.58 and 0.63 respectively. The global interobserver agreement and reliability were moderate to good (PA=0.82, K=0.63). We did not find a significant difference in the success rate between the different professions or according to the number of years of experience. In contrast, we found a much lower success rate on cases with a moderate hypoxia (pH between 7.05 and 7.20). These ambiguous cases are often associated with non-reassuring CTG patterns.

The main strength of the study is the large size and diversity of our sample which reflects the composition of a labor ward team. Also, the annotation tool developed for this study was appreciated by the participants and enabled to evaluate consistently how practitioners interpret CTG signals and the main clinical variables during delivery. The data on important characteristics of the participants (their profession, place of work and number of years of experience) enabled us to analyze how these characteristics impacted the success rate. Finally, our choice to include equal numbers of normal as pathological cases (pH lower than 7.15) was important to ensure that the participants annotated a sufficient number of cases with fetal hypoxia, which helped in estimating sensitivity and specificity with a high precision. Participants were not informed of the study design in which we presented the cases in batches of 10 CTGs randomly presented inside each batch with a 50/50 ratio of pathological and normal cases. It is very unlikely that this pattern was identified by the participants and that their answers would have been modified accordingly.

Our study adheres to the GRRAS guidelines [17], which are not followed by many similar studies according to Engelhart et al. [15]. We assessed agreement and reliability with PA and kappa respectively, as recommended by both the GRRAS guidelines [17] and the work by Costa Santos et al. [25] reviewing how agreement and reliability studies in obstetrics and gynecology should be conducted. Nevertheless, we identified some limitations. First, there is probably a selection bias in the participants included in the study. They are practitioners who voluntarily dedicated a substantial amount of time to annotating the cases. They may also be individuals who spend more time in the delivery room and are thus interested in taking part in studies evaluating CTG interpretation. Additionally, most participants were working in a university hospital, which may not be fully representative of the current demographics of maternity wards. These factors could contribute to the high level of agreement within this particular cohort, and lead to an overestimation of the accuracy compared to a general population of practitioners. Also, we made the choice to set the pH threshold corresponding to fetal hypoxia at 7.15. This enabled us to compare our results as accurately as possible with the existing literature, in particular with Hruban et al. [14] who used the same threshold as well as cases extracted from the CTU-UHB database. The pH 7.15 threshold corresponds to a moderate fetal hypoxia: in clinical practice, detecting it before it turns to a severe hypoxia gives practitioners the ability to intervene in a timely manner and ultimately leads to better outcomes. A more realistic setting would have been to define three CTG tracing categories (pathological, suspicious, normal) or even more, in accordance with the CTG interpretation guidelines [29]. However, this choice would have made comparison with existing literature more difficult. Finally, the CTG signals in the CTU-UHB dataset contain an important share of missing data points compared to other existing datasets: for example, there are in average 19% missing points in the FHR signal compared to 7% in the SPaM dataset [30]. Also, it is known that the FHR signal can be contaminated by the maternal heart rate [31]. These factors make CTG interpretation harder for practitioners [32, 33].

The results obtained in our large study confirm the limitations of visual interpretation of CTG signals with a low success rate, sensitivity and specificity. The comparison of our results with the literature evaluating CTG interpretation is challenging because existing studies generally have several differences including the choice of the classification system employed, the number of professionals involved in the study, the expertise or experience of the participating professionals, the multicenter design of the study, the specific pH threshold selected for defining hypoxia, and the statistical methods used to compute agreement and reliability. While our choice in using the group-level consensus rating per case offered simplicity in analyzing interprofessional agreement and reliability, this came with the risk of overestimating the measurement especially when compared to individual-level assessments. The existing study with the most similar protocol was Hruban et al [14], and we have been able to compare the annotations provided by the nine experts included in their study with our results on the same set of 100 cases. The success rate is comparable, but the experts have a higher specificity and lower sensitivity. Generally, experts have a better sensitivity than the general population [13, 34, 35], which may be consistent with their role, ultimately being a second line that assists in making decisions regarding a suspicious case. The design of our study, which includes as many normal cases as cases of fetal hypoxia, may explain the differences observed in the experts’ sensitivity. This highlights the challenging task of defining an expert in CTG interpretation.

We did not find a significant impact of the level of experience or the profession. Even if the difference is not significant, midwives had a better mean success rate in our cohort. This may be because all midwives that participated to the study practice daily in the labor ward, which may not be the case for some obstetrician-gynecologists (for example for those specialized in surgery). Also, as the midwives labelled in average more cases than the other professions, they may have improved their annotation skills with experience [36] using the feedback provided after each annotation. This trend may also be partly explained by them becoming more accustomed to the tool. Past studies involving both midwives and obstetricians are based on smaller or less diverse databases including only a few practitioners [10, 28, 34, 35, 37–40]. All of them found a poor interobserver reliability with a kappa coefficient ranging between 0.18 and 0.38. However, these studies only include a very small number of practitioners (less than ten), evaluated different outcomes, or had different inclusion criteria. For example, Blix et al. studied the assessment of CTG signals at admission [35], Figueras et al. included antepartum CTGs [40], Kundu et al. tried to predict the pH outcome from CTG signals [39] and Devoae et al. asked practitioners to annotate baselines, accelerations and decelerations but not to predict the fetal outcome [10]. Recently, a review by Engelhart et al. [15] did not find any clear association between the level of experience or profession and the accuracy of the annotations.

Finally, we found a higher success rate and stronger agreement for cases with a pH lower than 7.05 and for cases with a pH higher than 7.20. Inversely, cases with a pH between 7.05 and 7.20 were more challenging to annotate for our participants, with a success rate below 0.50 in this category. This conclusion is consistent with past studies [14, 35, 41, 42] and with a recent review highlighting the high reliability for CTG signals classified as normal [15]. In practice, when interpretation is difficult, some professionals use invasive second-line analyses to improve their ability to predict hypoxia, such as fetal scalp blood sampling (FBS) and ST analysis. While the interest of FBS remains a topic of debate [43], the contribution of STAN (ST Analysis) in retrospective cohorts has demonstrated its value in aiding clinical decision-making [44]. Our study showed that for ambiguous cases the practitioners’ success rate was indeed very low, confirming the need for specific tools to assist them. Beyond invasive analyses, computerized systems hold promising potential for improving the interpretation of CTG signals [45] and represent an interesting way to increase the accuracy while reducing interobserver variability [38, 46–48], especially within the critical pH range between 7.05 and 7.20.

## Conclusion

While the effectiveness of cardiotocography in reducing neonatal morbidity is still debated [49], it remains the primary method for assessing fetal well-being during labor. Several past studies have highlighted the poor accuracy of practitioners and the high interobserver variability in the interpretation of CTG signals. The use of an online annotation tool enabled us to gather the largest and most comprehensive database to evaluate the interobserver agreement and reliability in the interpretation of CTG signals.

We have shown that there is no significant difference in success rate between the different professions or levels of experience. Additionally, the cases with moderate hypoxia (pH between 7.05 and 7.20) were much harder to annotate with a mean success rate below a random guess. The possible selection biases in the participants of the study may even have overestimated the success rates and agreements in our cohort, and these results should be considered keeping in mind the complexity and pitfalls of agreement and reliability studies. As described in previous studies, we think that computerized systems helping practitioners in the interpretation [45] of CTG signals is a promising way to increase the accuracy while reducing interobserver variability in the future [38].

Also, the annotation tool developed as part of this research will lead to future studies. First, the continuous growth in the number of participants and annotations will make the results more robust and could enable to derive new insights. Second, the tool can be used to investigate specific questions, for example comparing the success rate of practitioners in different countries using different classifications, deepening our understanding of the cases that are hard to annotate for practitioners, or evaluate how the information provided by a computerized CTG system may assist them. Finally, it can also be used by practitioners as a training tool.

### Supplementary Information

**Additional file 1:** **Figure S1*.*** Annotation tool - Example of case labelling. **Figure S2.** Number of cases labelled per participant (sorted by number of labeled cases). **Figure S3.** Success rate as a function of the number of years of experience*.*

## Data Availability

On request, data and materials could be shared for research purpose please contact corresponding author at imane.benmbarek@aphp.fr.

## Abbreviations

CTG: Cardiotocography
FBS: Fetal blood sample
FHR: Fetal heart rate
FPR: False positive rate
HIE: Hypoxic-ischemic encephalopathy
PA: Proportion of agreement
SPaM: Signal processing and monitoring in labour
TPR: True positive rate
UC: Uterine contraction
