# Supplementary material for: Large-scale analysis of interobserver agreement and reliability in cardiotocography interpretation during labor using an online tool
Source: BMC Pregnancy Childbirth. 2024 Feb 14;24:136. doi: 10.1186/s12884-024-06322-4 (PMC10865637; doi:10.1186/s12884-024-06322-4)
Supplement: Supplementary file 1 — Additional file 1: Figure S1. Annotation tool - Example of case labelling. Figure S2. Number of cases labelled per participant (sorted by number of labeled cases). Figure S3. Success rate as a function of the number of years of experience. [file 12884_2024_6322_MOESM1_ESM.docx]

Supplemental data

Cardiotocography interpretation during labor by obstetric professionals: a large-scale online survey


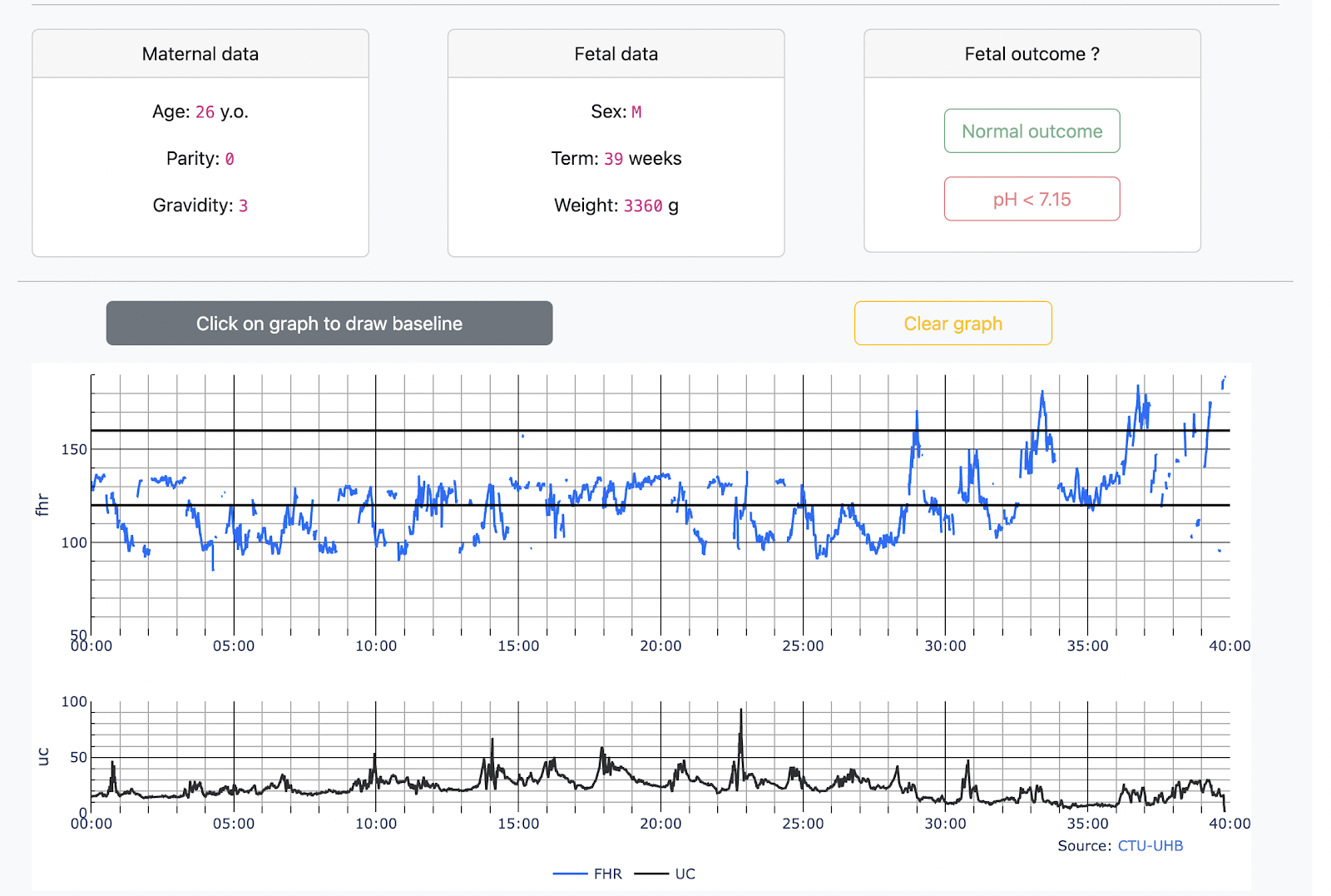


Figure S 1 Annotation tool - Example of case labelling


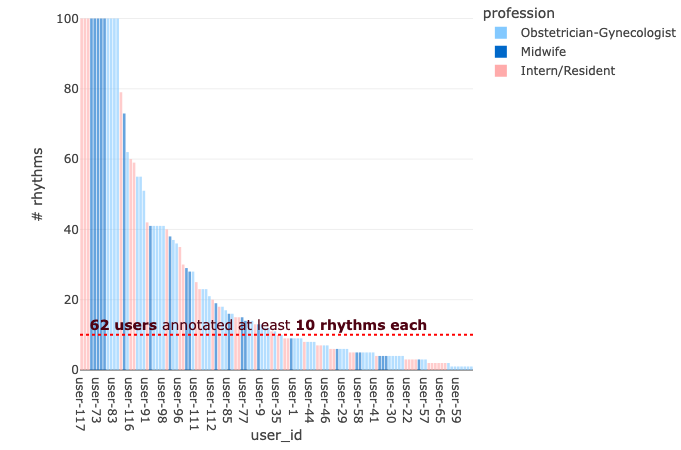


Figure S 2 Number of cases labelled per participant (sorted by number of labeled cases)


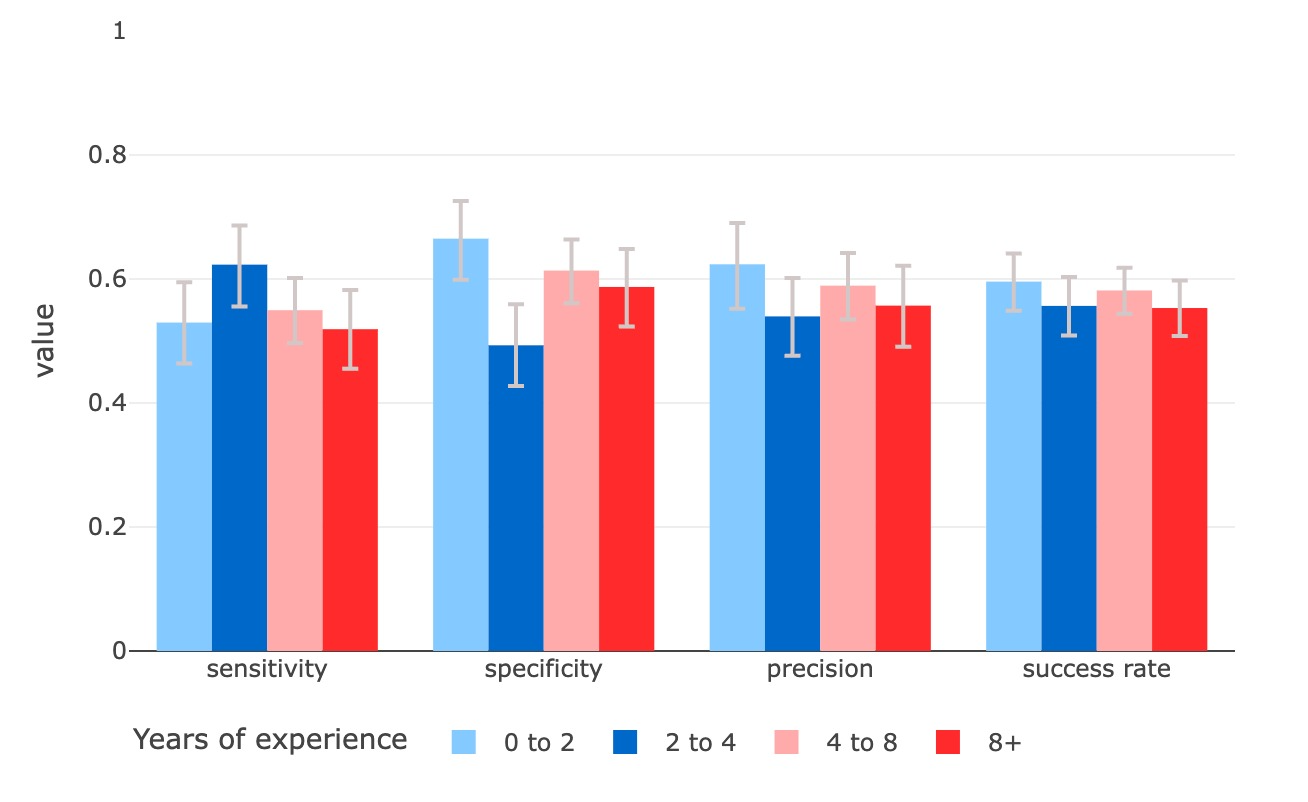


Figure S 3 Success rate as a function of the number of years of experience
